# A Software-Defined Sensor System Using Semantic Segmentation for Monitoring Remaining Intravenous Fluids

**DOI:** 10.3390/s25103082

**Published:** 2025-05-13

**Authors:** Hasik Sunwoo, Seungwoo Lee, Woojin Paik

**Affiliations:** Department of Computer Engineering, Konkuk University Glocal Campus, 268 Chungwon-daero, Chungju-si 27478, Chungcheongbuk-do, Republic of Korea; sunwoo@kku.ac.kr (H.S.); komsawa@naver.com (S.L.)

**Keywords:** smart healthcare, deep learning, semantic segmentation, PSPNet, medical image processing, IV fluid monitoring, software-defined sensors, real-time computer vision

## Abstract

**Highlights:**

**What are the main findings?**

- A smartphone-compatible computer vision system utilizing PSPNet delivers the highly accurate segmentation of IV fluid containers and fluid levels using only image data.
- The proposed system outperforms general-purpose segmentation models, such as the SAM, mainly when dealing with transparent medical containers.

**What are the implications of the main finding?**

- Enables low-cost, sensorless, real-time IV fluid monitoring in clinical and home-care settings, reducing nursing workload and improving patient safety.
- Demonstrates the feasibility of software-defined sensing in medical applications, replacing traditional hardware-based IV fluid sensors with deep learning solutions.

**Abstract:**

Accurate intravenous (IV) fluid monitoring is critical in healthcare to prevent infusion errors and ensure patient safety. Traditional monitoring methods often depend on dedicated hardware, such as weight sensors or optical systems, which can be costly, complex, and challenging to scale across diverse clinical settings. This study introduces a software-defined sensing approach that leverages semantic segmentation using the pyramid scene parsing network (PSPNet) to estimate the remaining IV fluid volumes directly from images captured by standard smartphones. The system identifies the IV container (vessel) and its fluid content (liquid) using pixel-level segmentation and estimates the remaining fluid volume without requiring physical sensors. Trained on a custom IV-specific image dataset, the proposed model achieved high accuracy with mean intersection over union (mIoU) scores of 0.94 for the vessel and 0.92 for the fluid regions. Comparative analysis with the segment anything model (SAM) demonstrated that the PSPNet-based system significantly outperformed the SAM, particularly in segmenting transparent fluids without requiring manual threshold tuning. This approach provides a scalable, cost-effective alternative to hardware-dependent monitoring systems and opens the door to AI-powered fluid sensing in smart healthcare environments. Preliminary benchmarking demonstrated that the system achieves near-real-time inference on mobile devices such as the iPhone 12, confirming its suitability for bedside and point-of-care use.

## 1. Introduction

Intravenous (IV) fluid therapy is a cornerstone of modern healthcare, widely used for hydration, medication delivery, and maintaining electrolyte balance in patients [1]. Despite its routine nature, errors in IV fluid administration—such as incorrect flow rates or unnoticed depletion—can pose serious risks, including medication misdelivery and patient harm. Studies have indicated that IV-related mistakes account for many nursing errors, often resulting from high workload and calculation inaccuracies [2,3].

Conventional methods for IV fluid monitoring primarily rely on weight-based or optical sensing systems. While weight-based systems provide accurate measurements by continuously tracking the fluid container mass, they require dedicated load cells and hardware, limiting their scalability and increasing their maintenance costs. On the other hand, optical methods determine the fluid volume through light refraction but often struggle with transparent and irregular container designs, reducing their reliability in real-world settings [4,5,6,7].

Low-cost infusion alarms are commonly used in clinical practice to address these limitations. These devices detect abnormal drip rates or flow interruptions using optical or photoelectric sensors [8,9], providing timely alerts that help prevent incidents like air embolism or backflow. However, their scope is limited to flow monitoring within the IV tubing and does not include the direct measurement of the fluid level inside the container—an essential factor in proactive infusion management.

Recent advances in artificial intelligence (AI) and computer vision offer promising alternatives. Semantic segmentation, in particular, enables the precise pixel-wise classification of image regions, allowing the accurate delineation of IV fluid levels without needing physical sensors. This technique is especially valuable for handling transparent containers where traditional sensing methods fail.

A recent work by Zunair and Hamza [10] proposed a masked supervised learning (MSL) framework to improve the semantic segmentation performance, particularly under data-scarce conditions. By partially masking ground-truth annotations during training, their approach enhances generalization and robustness against label overfitting. While promising in biomedical segmentation tasks, MSL has yet to be validated for highly transparent fluid segmentation, where visual cues are subtle and domain adaptation is essential. In contrast, our study focuses on a domain-specific architecture and IV-dedicated dataset, tailored to address transparency, container shape variation, and real-time deployment in clinical practice.

This study proposes a vision-based IV fluid monitoring system that functions as a software-defined sensor. Utilizing the pyramid scene parsing network (pyramid scene parsing network (PSPNet, version 1.0), our method segments IV containers and fluid content from smartphone-captured images to estimate the remaining volume in real time. The system eliminates hardware dependency and provides a scalable, cost-effective solution suitable for hospitals, clinics, and home-care environments.

This study presents the system’s technical architecture, segmentation accuracy across different datasets, and comparative analysis against the general-purpose segment anything model (SAM). This study also discusses the system’s potential integration into clinical workflows and outlines future directions for enhancing real-world applicability.

## 2. Related Work

This section reviews conventional and emerging approaches for IV fluid monitoring, including sensor-based systems, commercial devices, and deep learning techniques. We highlight the limitations of existing methods and identify research gaps addressed by our proposed solution.

### 2.1. Traditional IV Fluid Monitoring Methods

Historically, IV fluid levels have been measured using weight-based or optical systems. Weight-based monitoring involves specialized sensors that track the mass of IV fluid containers to estimate volume [5,6]. Although highly accurate, these systems require expensive and non-portable hardware, making them difficult to deploy at scale.

Optical methods attempt to gauge fluid levels based on refractive changes as the liquid volume decreases [7]. However, these methods are sensitive to container transparency, shape variation, and lighting conditions, often limiting their accuracy and reliability in clinical environments.

Drip-based monitoring systems offer a lower-cost alternative, detecting flow disruptions using optical drop or photoelectric sensors [8,9]. These devices trigger alarms when the drip rate slows or stops, helping prevent adverse events such as air embolism. However, they do not directly estimate the fluid remaining in the IV container, leaving a critical monitoring gap unaddressed.

In addition to these conventional systems, the recent research has explored more advanced biomedical sensing technologies. For instance, reconfigurable multimode microwave sensors have been proposed for the non-invasive monitoring of biological fluids. Wang et al. introduced a microwave-based sensor that utilized resonance and transmission sensing modes to detect glucose concentration changes through variations in dielectric properties [11]. While their system was designed for glucose monitoring, the underlying sensing principles could be adapted for IV fluid estimation as well. These approaches offer non-contact measurement and high sensitivity, but their reliance on complex circuitry and specialized materials may present barriers to widespread clinical use.

Our study addresses these limitations by proposing a software-defined sensing approach that eliminates the need for physical sensors. By leveraging computer vision and deep learning, our system enables fluid volume estimation using only image data, providing a low-cost and scalable alternative to traditional sensor-based methods.

### 2.2. Commercial IV Monitoring Devices

Several commercial products have been developed to support IV fluid management, such as Shift Labs’ DripAssist, Monidor’s Monidrop, and Evelabs’ Dripo. These devices clip onto IV tubing and monitor flow rates in real time [12,13,14]. While effective in detecting drip anomalies, they require external sensors. They are not designed to assess the actual fluid volume inside the container—their reliance on hardware limits adaptability across diverse container types and non-standard clinical settings.

### 2.3. Computer Vision in Medical Applications

Recent studies have explored computer vision as an alternative to hardware-based IV monitoring. For instance, using basic convolutional networks, one approach used an Arduino Portenta H7 with Vision Shield to classify the IV fluid levels into three discrete categories [15]. While the system achieved reasonable accuracy, it lacked fine-grained fluid estimation and required dedicated hardware.

In laboratory settings, segmentation models such as mask R-CNN and fully convolutional networks (FCNs) have been applied to recognize materials and vessels in structured environments [16]. These models performed well under controlled conditions but struggled with transparency and irregular shapes, making them less suitable for IV fluid monitoring.

Semantic segmentation, by contrast, enables dense pixel-wise classification, which is well suited to identifying the exact fluid boundaries in IV containers. The use of such methods in vision-based sensing eliminates the need for physical sensors and allows for flexible, low-cost deployment across a variety of clinical contexts.

Beyond segmentation, the recent research has also highlighted the importance of structured scene understanding for downstream tasks such as automated reasoning and status recognition. For example, scene graph generation techniques can extract and model relationships between visual entities in an image, enabling systems to understand the overall clinical context. Han et al. [17] proposed a divide-and-conquer predictor for generating unbiased scene graphs, which preserves object relationships and mitigates label imbalance in complex scenes. Integrating such methods into our framework may support future downstream applications such as clinical status inference, automated reporting, or triggered alerts based on the interaction between IV containers, tubing, and patient activity.

### 2.4. Advances in Semantic Segmentation

Semantic segmentation enables dense pixel-wise classification, making it ideal for transparent fluid applications. Among the leading models, the pyramid scene parsing network (PSPNet) combines ResNet-based feature extraction with pyramid pooling to capture both local details and global context [18]. PSPNet has achieved high accuracy in general datasets such as PASCAL VOC and has shown promise in specialized domains, including laboratory vessel segmentation [16,18].

Other recent segmentation models, including FFTI [19], DARGS [20], and MFFN [21], introduce attention and feature fusion mechanisms to enhance performance. Additionally, lightweight architectures such as BiSeNet V2 [22] and transformer-based SegFormer [23] offer faster inference suitable for mobile and embedded systems—making them attractive options for future deployment.

General-purpose models like the segment anything model (SAM) [24] offer versatility across image domains using transformer-based segmentation guided by prompts. However, studies have shown that the SAM underperforms in segmenting transparent medical fluids, often requiring manual threshold tuning and struggling with visual ambiguity [25,26]. In contrast, task-specific models trained on domain-relevant datasets typically offer superior segmentation accuracy in medical contexts.

### 2.5. Gaps and Contributions

Despite technological progress, current solutions still exhibit key limitations:

- Traditional sensors and commercial monitors do not provide the direct volume estimation of IV fluids.
- Many vision-based methods lack granularity or are constrained by hardware requirements.
- General-purpose segmentation models struggle with the transparency and reflectivity of IV containers.

This study addresses these challenges by introducing a vision-based, software-defined sensing system using PSPNet for real-time IV fluid monitoring. Our approach eliminates the need for dedicated hardware, leverages domain-specific training, and achieves high segmentation accuracy even for transparent containers. It bridges the gap between flow-based monitoring and container-level volume estimation—advancing the integration of AI in healthcare sensing technologies.

## 3. Materials and Methods

This section details the core technical components of the proposed IV fluid monitoring system, including the segmentation approach, model architecture, system design, dataset construction, and implementation.

### 3.1. Overview of Semantic Segmentation

**Figure 1:**
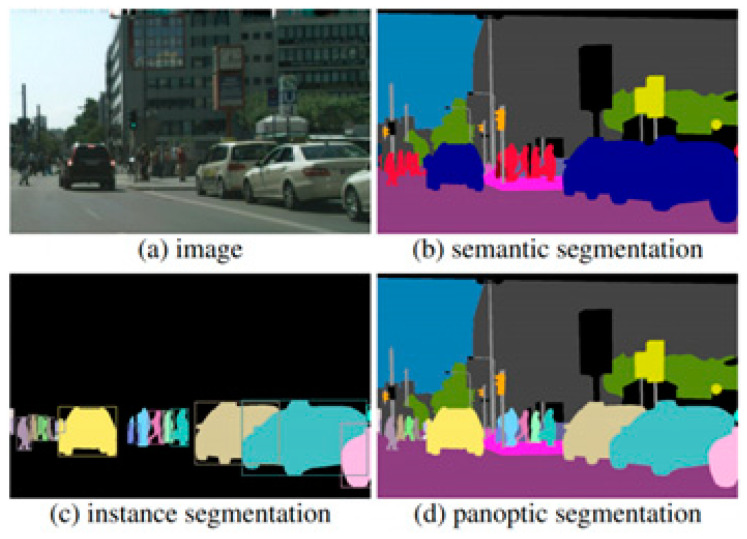
Comparison of segmentation methods: semantic, instance, and panoptic segmentation.

Semantic segmentation was selected for this task due to its ability to classify each pixel in an image, which is critical for estimating the volume of transparent fluids within medical containers. Unlike object detection or classification, semantic segmentation provides the granularity required to distinguish subtle transitions between fluid and air, or between container edges and background. Figure 1 illustrates the differences between segmentation approaches and highlights the precision enabled by semantic segmentation in our use case.

However, not all segmentation models are equally effective for medical imaging, especially when handling challenges such as transparency, reflection, and low-contrast fluid boundaries. General-purpose models often fail to address these issues without extensive post-processing. To address this, we adopt the pyramid scene parsing network (PSPNet)—a state-of-the-art semantic segmentation model known for its ability to incorporate both fine details and the global context through multi-scale feature aggregation.

The following section introduces the core architecture of PSPNet and details of how we customized it to build a fluid segmentation system optimized for IV container imagery captured using consumer-grade smartphones.

### 3.2. PSPNet Architecture for Fluid Segmentation

The pyramid scene parsing network (PSPNet) is a deep learning architecture designed for semantic segmentation [18], which can capture both global and local contextual information. This study uses PSPNet to identify and segment IV fluid regions and container boundaries in images captured by smartphones. The model comprises the following three stages:

1. Feature Extraction (Backbone: ResNet-50): PSPNet uses ResNet-50 as its backbone for feature extraction. This version of ResNet was selected as a practical trade-off between segmentation accuracy and computational efficiency, which is essential for real-time deployment on smartphones and embedded devices. Table 1 summarizes the performance comparison between ResNet-50 and a deeper alternative, ResNet-101.
2. Pyramid Pooling Module (PPM): The ResNet-generated feature maps are passed into the PPM, which performs pooling at multiple spatial scales (e.g., 1 × 1, 2 × 2, 3 × 3, 6 × 6). These pooled features are upsampled and concatenated with the original map to capture both local details and the global context—critical for segmenting transparent fluids in complex clinical settings.
3. Segmentation Prediction: The integrated multi-scale features are processed through convolutional layers and upsampled using bilinear interpolation to match the input resolution. This allows the model to output dense segmentation maps distinguishing two classes: Vessel and Liquid General.

The PSPNet architecture is particularly effective for this application because it addresses the following key challenges in medical image segmentation:

- The need to distinguish transparent and reflective surfaces;
- Handling variations in container size and shape;
- Segmenting fluid that may have weak visual boundaries.

While PSPNet delivers high segmentation accuracy, it is computationally heavier than lightweight architectures like BiSeNet V2 or SegFormer, better suited for real-time use on smartphones or embedded devices [22,23]. Although our current focus is accuracy, future work will explore such models for broader clinical deployment.

### 3.3. Model Architecture Components

**Figure 2:**
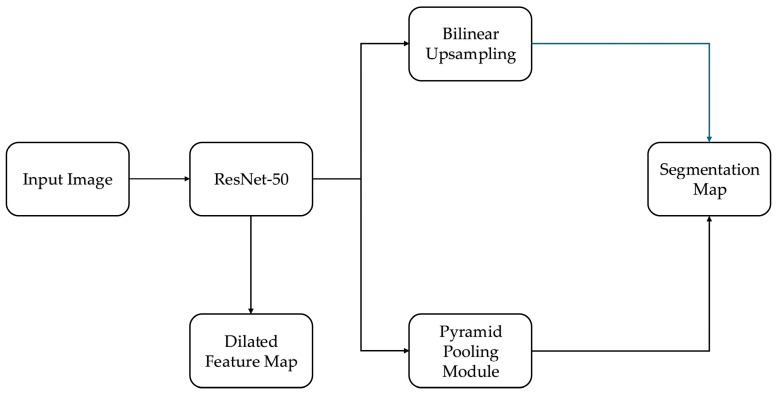
Architecture of the PSPNet-based segmentation model with ResNet-50 backbone, pyramid pooling, and domain-specific enhancements for IV fluid segmentation.

The proposed segmentation model builds on the foundational structure of fully convolutional networks (FCNs), which enable pixel-level predictions by replacing dense layers with convolutional layers. However, to meet the precision and efficiency demands of IV fluid monitoring, we introduce several architectural enhancements inspired by and extending the canonical PSPNet [18]. The full architecture is illustrated in Figure 2.

The key components and innovations include:

1. Skip Connections (ResNet-50): The model uses a residual architecture with skip connections, allowing the network to preserve both the low-level and high-level spatial features essential for the accurate boundary detection of transparent fluids.
2. Dilated Convolutions: Within the ResNet backbone, dilated convolutions expand the receptive field without reducing feature resolution. This enables the network to capture a wider context, helping it to determine fluid regions that lack clear edges or contrast.
3. Pyramid Pooling Module (PPM): While standard FCNs lack context aggregation, our model includes a multi-scale pooling module that enriches the feature map with local and global spatial contexts. This improves the model’s ability to handle irregular IV container shapes and occlusions, which are common in real-world clinical scenarios.
4. Efficient Upsampling: Instead of the transposed convolutions often used in FCNs, we use bilinear interpolation to upsample feature maps to the original input size. This method is both computationally efficient and less prone to checkerboard artifacts, preserving smooth boundaries between the vessel and fluid regions.
5. Clinical Adaptation of Canonical PSPNet: In contrast to the original PSPNet, which uses ResNet-101 and is trained on general-purpose datasets like PASCAL VOC, our model is optimized for domain-specific deployment. We use ResNet-50 to reduce inference time and memory usage for mobile compatibility (as detailed in Section 3.2). We fine-tune PSPNet exclusively on IV-specific data involving transparent fluids, irregular containers, and varied lighting, addressing segmentation challenges not considered in the canonical implementation.
  - These architectural choices—dilated residual encoding, pyramid-based context fusion, efficient upsampling, and task-specific training—enable a robust segmentation performance under real-world hospital conditions. The resulting system is both technically optimized and clinically relevant for software-defined sensing.

The FCN-style architecture, deep ResNet features, and pyramid-level context aggregation make PSPNet well suited for medical image segmentation tasks, especially those involving transparent, amorphous regions like IV fluids [27]. This combination of spatial precision and contextual awareness is critical for the accurate estimation of fluid volumes in sensorless monitoring applications.

### 3.4. System Design and Data Flow

The proposed IV fluid monitoring system uses consumer-grade mobile devices for practical deployment. A smartphone, operated by a nurse or caregiver, captures an image of the IV fluid container in a clinical setting such as a hospital room or nursing station.

Once captured, the image is transmitted to a cloud-based or local processing server, depending on the deployment configuration. The server runs the trained PSPNet segmentation model, which processes the input and produces a segmentation mask with two classes:

- Vessel (the IV container boundary);
- Liquid general (the fluid region inside).

The system then estimates the remaining fluid volume by computing the pixel ratio of the segmented fluid region relative to the total vessel region. This ratio provides a practical, sensorless proxy for volume estimation and can be further calibrated based on container geometry. This architecture enables the following:

- Non-contact, low-cost monitoring using standard mobile hardware;
- Flexible deployment either locally at the bedside or via the centralized hospital infrastructure;
- Integration into broader healthcare automation systems such as nurse robots or patient monitoring dashboards.

The system design focuses on scalability, simplicity, and ease of use, minimizing the need for specialized equipment or training.

### 3.5. Dataset Creation and Implementation Details

To train and validate the proposed segmentation model, we created an IV-specific dataset by collecting 2636 smartphone-captured images of IV containers, supplemented with 370 online-sourced images to increase the container diversity. Annotation masks were manually generated using the Labelme tool, an open-source image labeling software designed for pixel-wise annotation tasks [28]. The labeling schema included two classes, i.e., *Vessel* and *Liquid General*, enabling precise boundary definitions even for transparent or reflective fluid surfaces.

The annotation process involved outlining the vessel exterior and the internal fluid surface separately for each image. Annotated masks were exported in the JSON format and subsequently converted into segmentation masks compatible with PyTorch (v1.13) training pipelines.

In addition to the IV-specific dataset, we used the publicly available LabPics dataset [16], which contains material and vessel segmentations from general laboratory scenes. To enhance generalization, a third training set (“Mixed”) was constructed by combining the IV-specific dataset and the LabPics dataset.

The model was trained with the following hyperparameters: a learning rate of 0.0001, a batch size of 16, and a total of 500,000 iterations. Stochastic gradient descent (SGD) was used as the optimizer, with a momentum coefficient of 0.9. A step decay learning-rate schedule was applied, reducing the learning rate by a factor of 0.1 every 150,000 iterations to stabilize convergence.

The cross-entropy loss function was used for pixel-wise classification. To improve generalization and robustness, data augmentation techniques were employed, including random horizontal flipping, brightness variation within ±15%, and random cropping to 90% of the original image resolution. Early stopping was not applied, as training proceeded for a fixed number of iterations based on validation loss stability.

All experiments were implemented using PyTorch (v1.13) and trained on an NVIDIA RTX A5000 GPU (NVIDIA Corporation, Santa Clara, CA, USA) with 24 GB of VRAM. The input resolution for training was set to 1920×1080 pixels. Inference benchmarking was additionally performed on an iPhone 12 (Apple Inc., Cupertino, CA, USA) and a Raspberry Pi 4B (Raspberry Pi Foundation, Cambridge, UK) with TPU acceleration, as detailed in Section 3.6.

The trained model achieved stable convergence without signs of overfitting, as evidenced by the plateauing validation loss and consistent mIoU scores across multiple test subsets.

### 3.6. Inference Time Evaluation on Mobile Platforms

To assess the feasibility of deploying the proposed segmentation model in real-world healthcare environments, we conducted preliminary inference benchmarking on two representative resource-constrained platforms: an iPhone 12 (Apple A14 Bionic chip) and a Raspberry Pi 4B (8GB RAM) with a Google Coral USB TPU (Google LLC., Mountain View, CA, USA). The trained PSPNet model with a ResNet-50 backbone was exported to the ONNX format and optimized for mobile inference using Core ML Tools (iOS) and TensorRT Lite (embedded).

On the iPhone 12, the system achieved an average inference time of 275 ms per image (full HD resolution, 1920 × 1080), enabling near-real-time monitoring at approximately 3–4 frames per second (FPS). On the Raspberry Pi 4B with TPU acceleration, the model processed one frame in 480 ms, corresponding to 2 FPS, under standard indoor lighting conditions.

These results demonstrate the practical deployability of the proposed model on modern smartphones and embedded platforms, particularly for point-of-care or bedside applications. To further improve inference speed and energy efficiency, future work will evaluate lightweight architectures such as BiSeNet V2 and SegFormer, which offer reduced model complexity while maintaining high segmentation accuracy.

## 4. Evaluation Criteria

Evaluation is critical for developing a robust computer vision system for IV fluid monitoring. This section outlines the metrics and methodologies used to assess the proposed model’s performance, incorporating original experiments and a comparative analysis with recent state-of-the-art models, including the segment anything model (SAM) [24]. All metrics are carefully chosen to evaluate the segmentation quality and practical applicability comprehensively.

### 4.1. Metrics for Evaluation

**Figure 3:**
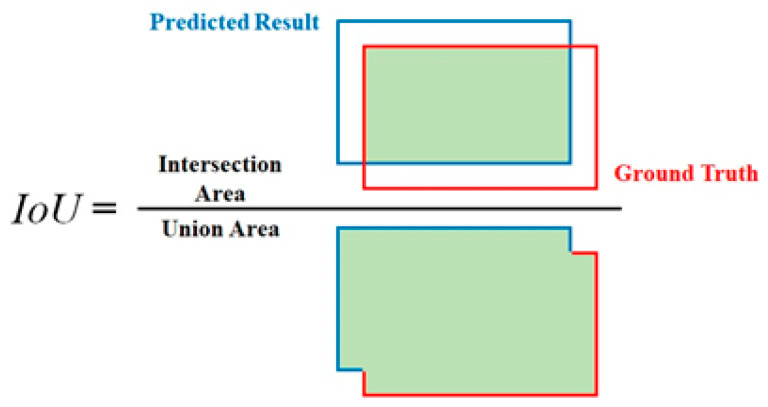
Definition of intersection over union (IoU), used as primary metric for segmentation accuracy.

Semantic segmentation requires metrics that accurately quantify the overlap and alignment between predicted outputs and ground truth. In this study, we used the following evaluation metrics:

- Mean Intersection over Union (mIoU): mIoU is the primary metric for semantic segmentation, calculated as the average IoU across all classes [29]. IoU measures the overlap between the predicted segmentation and the ground truth, as shown in Figure 3. Higher IoU values indicate better alignment. A prediction is considered correct if the IoU exceeds a threshold of 0.5, based on the standards of the PASCAL VOC object recognition challenge and COCO dataset criteria [30].

(1) $$IoUc=\frac{True\;Positive\;(TP)}{True\;Positive\;(TP)+False\;Positive\;(FP)+False\;Negative\;(FN)},$$

(2) $$mIoU=\frac{1}{N}\sum_{c=1}^{N}IoUc$$

The IoU is defined as the ratio of the overlap between the predicted segmentation and the ground truth to the total union of both for a given class *c*, and we take the average of the IoU values across all *N* classes to calculate the mean intersection over union (mIoU).

- Precision and Recall: Precision evaluates the proportion of correctly predicted positive pixels among all predicted positive pixels, while recall assesses the proportion of true positives (TPs) identified among all actual positives [31]. These metrics complement mIoU by providing insight into the balance between false positives (FPs) and false negatives (FNs).

(3) $$precision=\frac{TP}{TP+FP}$$

(4) $$recall=\frac{TP}{TP+FN}$$

**Figure 4:**
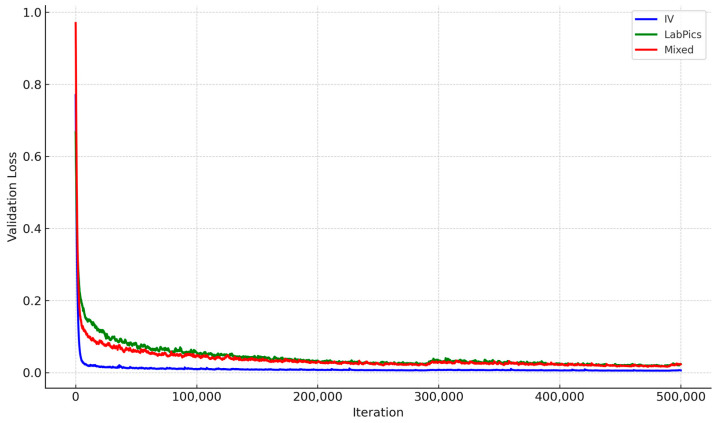
Validation loss trends during training for Liquid General segmentation across datasets. IV-specific model shows faster convergence and more stable loss.

- Validation Loss: The loss values during the validation phase, as shown in Figure 4, track the model’s convergence and overfitting tendencies over training iterations.

### 4.2. Dataset-Specific Comparison

The model was evaluated on three datasets. The IV dataset is a dedicated dataset of IV container images captured under realistic conditions. It represents the proposed system’s primary test case. The LabPics dataset is a dataset of general laboratory vessel images adapted for this study to evaluate the model’s performance in a broader context. The mixed dataset combines the IV and LabPics datasets to assess the impact of dataset diversity on model performance. Table 2 provides a detailed breakdown of the data distribution across each dataset’s training, validation, and test sets.

**Figure 5:**
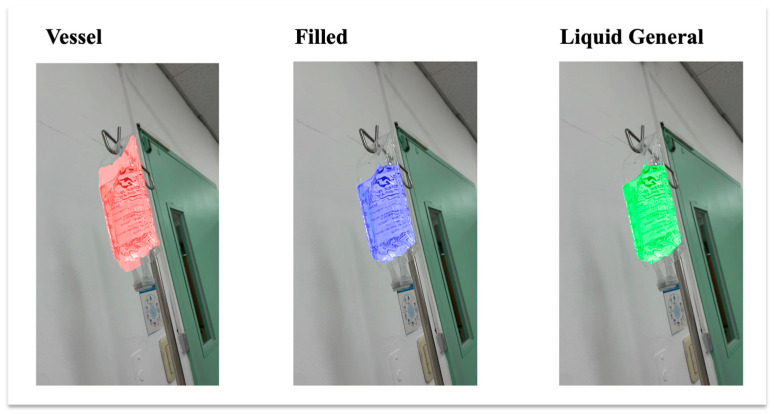
Example of IV fluid containers showing three object types. Filled and Liquid General are treated as the same class in this study, with only Liquid General used for segmentation.

The baseline dataset was the LabPics dataset developed for recognizing materials and vessels in chemistry lab settings. According to the LabPics dataset definition, the chemistry vessel images included objects classified as Vessel, a container; Filled, which refers to all content in the container; and Liquid General, which is the liquid-type content [16]. In this study, we regard Filled and Liquid General as the same since the IV fluid is mainly liquid with some viscosity differences. Thus, we semantically segmented only Vessel and Liquid General. Figure 5 shows the object types in the IV containers.

### 4.3. Model Performance

**Figure 6:**
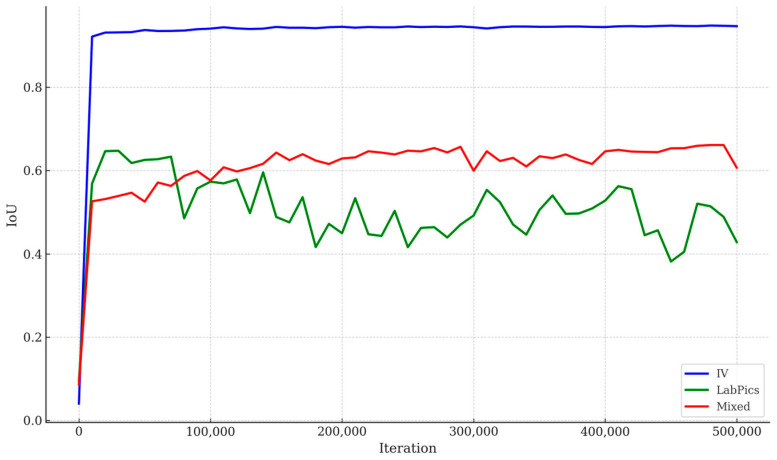
IoU trends over training iterations across IV-specific, LabPics, and mixed datasets. IV-specific model consistently achieved higher pixel-level segmentation accuracy during training, indicating effectiveness of domain-specific dataset adaptation for IV fluid container segmentation.

Figure 5 illustrates the validation loss for segmenting the Liquid General region across the three datasets. The IV-specific model consistently outperformed the others, with lower validation loss and more stable convergence. Figure 6 shows the pixel-level IoU trends during training, where the IV-specific model demonstrated superior performance. Table 3 summarizes the mIoU values for Vessel and Liquid General areas based on test data, confirming that focused training on IV-specific data yields the best results.

### 4.4. Comparative Analysis with SAM

**Figure 7:**
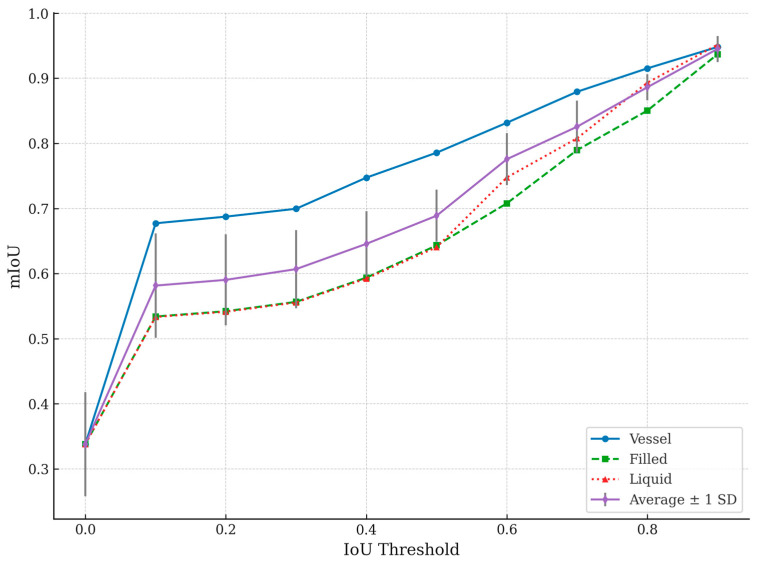
Segment anything model (SAM) performance evaluated across different IoU thresholds for Vessel, Filled, and Liquid General segmentation classes. Average mIoU across all classes is also plotted, with error bars representing ±1 standard deviation across test samples. Vessel segmentation achieves higher accuracy compared to Filled and Liquid General regions, particularly at lower thresholds.

A comparative experiment was conducted to validate the robustness of the proposed method using the segment anything model (SAM) with the UnSAM+ variation [25]. The SAM is a transformer-based segmentation model known for its adaptability and generalization across diverse tasks [24]. The IV dataset was used to ensure consistency with the proposed model’s evaluation for this experiment. Regarding the metrics, mIoU, precision, and recall were calculated for the SAM at various IoU thresholds, with the results visualized in Table 4 and Figure 7.

The SAM demonstrated an average mIoU of 0.33 across all classes without input points but required thresholding to improve performance, as outlined in Table 4. The proposed PSPNet-based model outperformed the SAM significantly regarding mIoU for both the Vessel and Liquid General classes. This result highlights the advantage of task-specific training and dataset optimization over general-purpose models.

Table 4 presents how the SAM’s segmentation performance improves with increasing IoU thresholds, highlighting its limitations and strengths compared to the proposed model. These trends are also visualized in Figure 7, which plots the mIoU values for the Vessel, Filled, and Liquid General classes, along with the average mIoU curve and associated variability. The threshold column represents the minimum IoU required for a segmented area to be considered accurate. Increasing the threshold makes the evaluation stricter, requiring a higher overlap between the predicted segmentation and ground truth. The Vessel mIoU column shows the IoU score for detecting the IV fluid container (Vessel). A higher value indicates the better segmentation of the container. The Liquid mIoU column reveals IoU for segmenting the Liquid General region of the IV fluid (i.e., the part occupied by liquid). This observation is crucial for estimating fluid volume accurately. The average mIoU shows the overall mean IoU across all three segmentation categories, representing the model’s general segmentation performance.

At a threshold of 0.0, all IoU values are 0.3382 across the Vessel and Liquid General categories. This result indicates that the SAM struggled with accurate segmentation when evaluated with minimal constraints.

Between the thresholds of 0.0 and 0.4, the average mIoU increases from 0.3382 to 0.6457. The Vessel mIoU improves significantly, reaching 0.7474 at a threshold of 0.4. However, the Liquid General classes show slower improvement, hovering around 0.59, indicating difficulty in accurately segmenting fluid regions.

From the threshold of 0.5 to 0.7, the average mIoU surpasses 0.82, showing that the SAM performs better under stricter conditions. The Vessel mIoU exceeds 0.87, meaning the container is being correctly segmented more often. The Liquid General mIoU values approach 0.80, showing that the SAM is becoming more reliable for fluid segmentation.

At thresholds between 0.8 and 0.9, Vessel segmentation reaches 0.9481 at a threshold of 0.9, meaning the model is highly accurate in detecting the IV container. The Liquid General regions surpass 0.95, suggesting that the SAM performs reasonably well when a high IoU overlap is enforced. The average mIoU reaches 0.9451 at a threshold of 0.9, showing that the SAM can achieve near-perfect segmentation in some cases.

The SAM continuously improves as thresholds increase, indicating that it can accurately segment IV containers and fluids when adequately guided. It performs better at vessel segmentation than identifying general liquid regions, suggesting it is more effective at detecting container boundaries than distinguishing transparent fluids.

However, its improvement in liquid segmentation is slow, indicating difficulties with the transparency of IV fluids, which leads to partial segmentations. The model’s performance depends on tuning the threshold for optimal results, showing that it lacks consistent precision in medical-specific segmentation tasks. It tends to misidentify or incompletely segment IV fluid regions at lower thresholds unless additional input prompts are provided.

The PSPNet-based model demonstrated more stable and accurate segmentation across all categories without threshold tuning. In contrast, the SAM required threshold adjustments and showed inconsistent performance, while the PSPNet-based model achieved higher precision without extensive post-processing. Future work could explore integrating the SAM’s adaptability with PSPNet’s domain-specific precision to develop an optimized hybrid system.

As shown in Table 4 and Figure 7, the SAM performs well at high IoU thresholds but struggles with transparent fluid segmentation at lower thresholds—highlighting the challenges of applying general-purpose models to domain-specific medical tasks. The PSPNet-based approach outperforms the SAM for IV-specific tasks, emphasizing the importance of training with specialized datasets rather than relying on general-purpose segmentation models.

Figure 7 illustrates the segmentation performance of the segment anything model (SAM) across various IoU thresholds, separately showing results for the Vessel, Filled, and Liquid General classes. The plot also includes the average mIoU across all three classes, with error bars representing ±1 standard deviation across test samples. As the IoU threshold increased from 0.0 to 0.9, the segmentation performance improved steadily for all classes. Among the classes, Vessel segmentation consistently achieved the highest mIoU scores, followed by Filled, while Liquid General segmentation exhibited relatively slower improvement at lower thresholds. These results highlight that while the SAM demonstrates increasing segmentation consistency with stricter matching criteria, it performs better on solid and structured regions (such as vessel boundaries) than on transparent fluid regions.

The observed trends suggest that SAM’s general-purpose architecture, although adaptable, encounters challenges when segmenting transparent or amorphous objects like IV fluids, whereas rigid container structures are more reliably detected. In contrast, the PSPNet-based model presented in this study achieved more stable and consistently higher segmentation performance across all classes without requiring manual threshold adjustments.

### 4.5. Insights from Comparison with SAM

While SAM offers versatility and generalization, its performance on transparent and irregular IV containers was limited by factors such as partial vessel detection and interference from printed labels. The proposed model, trained specifically on IV datasets, overcame these challenges by focusing on domain-specific characteristics, demonstrating the importance of specialized training for medical applications.

### 4.6. Limitations and Further Validation

Although the proposed model outperformed the SAM, its dependency on IV-specific datasets may limit generalization to other fluid containers or environments. Future work could explore hybrid models combining the domain-specific precision of PSPNet with the adaptability of transformer-based architectures like the SAM.

## 5. Results

### 5.1. Model Performance Across Datasets

The proposed PSPNet-based IV fluid measurement system was evaluated on three datasets: the IV-specific dataset, the LabPics dataset, and the mixed dataset. The IV-specific dataset achieved the highest mIoU scores (0.94 for Vessel and 0.92 for Liquid General), indicating high performance in segmenting IV fluids (Table 3).

The LabPics dataset showed lower accuracy for fluid segmentation (mIoU = 0.40), highlighting challenges in generalizing different container shapes and transparency levels. The mixed dataset improved segmentation slightly (mIoU = 0.84 for Liquid General) but remained inferior to the IV-specific model.

### 5.2. Validation Loss and Training Stability

The IV-specific model exhibited faster convergence with lower final validation loss, confirming effective learning for IV container segmentation (Figure 4). In contrast, the LabPics model struggled with higher loss values, suggesting difficulty adapting to transparent fluid segmentation.

### 5.3. Segmentation Evaluation Metrics

To evaluate the segmentation performance of the proposed system, several complementary metrics were used, and they are as follows:

- Mean Intersection over Union (mIoU): measures the overlap between the predicted and ground-truth segmentation, averaged across vessel and fluid classes.
- Dice Coefficient: this provides a region-based overlap measure, calculated as twice the area of overlap divided by the sum of pixels in the prediction and ground-truth masks.
- Pixel-wise F1-Score: this computes the harmonic mean of precision and recall for each class, offering a balanced view of segmentation accuracy.
- Hausdorff Distance (Future Work): Although not included in the current experiments, Hausdorff distance is a useful metric to assess boundary agreement between predicted and ground-truth masks. Future work will incorporate Hausdorff distance analysis to evaluate fine boundary delineation.

Table 5 summarizes the performance of the IV-specific PSPNet model across these metrics. The high dice coefficient (0.965) and F1-score (0.955) values confirm that the model achieves not only a high overall overlap (mIoU = 0.93) but also robust class-wise precision and recall.

### 5.4. Comparative Analysis with Segment Anything Model (SAM)

The segment anything model (SAM) was evaluated using the IV dataset at various intersection over union (IoU) thresholds. As shown in Table 4 and Figure 7, the SAM’s performance improved significantly at higher thresholds (0.8–0.9), achieving an average mIoU of 0.9451. However, at lower thresholds (0.0–0.4), the segmentation performance was low (mIoU = 0.3382), particularly for transparent fluid regions. This threshold sensitivity indicates that the SAM’s automatic segmentation mode struggles to consistently detect subtle boundaries without explicit guidance.

In contrast, the PSPNet-based model consistently delivered higher segmentation accuracy without requiring post-processing or threshold adjustments. PSPNet proved more robust in handling the challenges of transparent fluid segmentation, such as weak fluid–air interfaces, container reflections, and lighting variations. This stability reinforces PSPNet’s suitability for clinical IV fluid monitoring, where fully automatic performance without human intervention is essential.

While this study focuses on comparing PSPNet with the SAM in terms of the automatic segmentation mode, we acknowledge that the SAM is primarily designed for prompt-based segmentation, utilizing user-provided points, boxes, or textual prompts. A comprehensive evaluation would involve assessing the SAM’s performance under prompt-driven conditions, where targeted inputs could potentially enhance segmentation accuracy. Future work will explore prompt-based comparisons to better assess the strengths of the SAM relative to domain-specific models like PSPNet, particularly in real-world clinical environments where ease of deployment and automation are critical.

Furthermore, while our analysis centered on PSPNet versus the SAM, we recognize the importance of broader benchmarking with lightweight or medical-focused segmentation models. Models such as SegFormer and BiSeNet V2 represent promising directions for improving the real-time performance on mobile and embedded platforms. Future benchmarking efforts will incorporate these architectures to more comprehensively analyze trade-offs between segmentation accuracy, model complexity, and inference efficiency.

### 5.5. IoU Trends and Precision–Recall Analysis

Figure 6 presents IoU performance trends, confirming that PSPNet trained on the IV dataset consistently achieved the highest segmentation accuracy. Precision–recall analysis showed the following:

- The IV-specific model maintained a recall of >90%, effectively detecting fluid regions;
- The LabPics model had a lower precision, leading to false positives in fluid classification.

### 5.6. Ablation Study

To better understand the contributions of individual architectural components and design choices to overall segmentation performance, an initial ablation analysis was conducted.

First, we compared two different backbones for feature extraction: ResNet-50 and ResNet-101. As reported in Table 1 (Section 3.2), ResNet-101 offered only a marginal improvement in segmentation accuracy (approximately +1% mIoU) while substantially increasing inference time and memory usage. This result confirms that the choice of a lighter ResNet-50 backbone offers a favorable balance between performance and deployability, especially for mobile applications.

Second, we evaluated the impact of the Pyramid Pooling Module (PPM) by comparing PSPNet’s full model against a variant without the PPM layer. Removing the PPM resulted in a decrease of approximately 4% in mIoU on the IV-specific test set, highlighting the importance of multi-scale context aggregation in effectively segmenting transparent fluid boundaries.

Third, to assess the influence of data augmentation, a training run without random brightness variation or cropping was performed. This model achieved slightly lower validation mIoU (~1.5% decrease) and demonstrated higher variance in fluid boundary predictions, suggesting that augmentation contributes to improved generalization under variable imaging conditions.

Although a comprehensive layer-by-layer ablation was beyond the current scope, these preliminary experiments demonstrate that multi-scale feature pooling and data augmentation are critical for achieving robust segmentation in clinical IV monitoring scenarios.

Future work will involve more detailed ablation studies, including the layer-specific feature contribution analysis and evaluation of alternative pyramid pooling strategies.

### 5.7. Fluid Volume Estimation Accuracy and Inference Time Validation

To validate the system’s ability to estimate fluid volumes based on segmentation outputs, a preliminary quantitative evaluation was conducted comparing the estimated fluid volumes against ground-truth measurements.

Ground-truth volumes were obtained by weighing IV fluid containers at different fill levels using a calibrated digital scale (resolution: 0.1 g) and converting mass to volume based on known fluid densities. Corresponding container images were processed through the trained PSPNet model to segment fluid regions. Estimated fluid volumes were calculated based on the proportion of segmented fluid pixels relative to the known container geometry.

**Figure 8:**
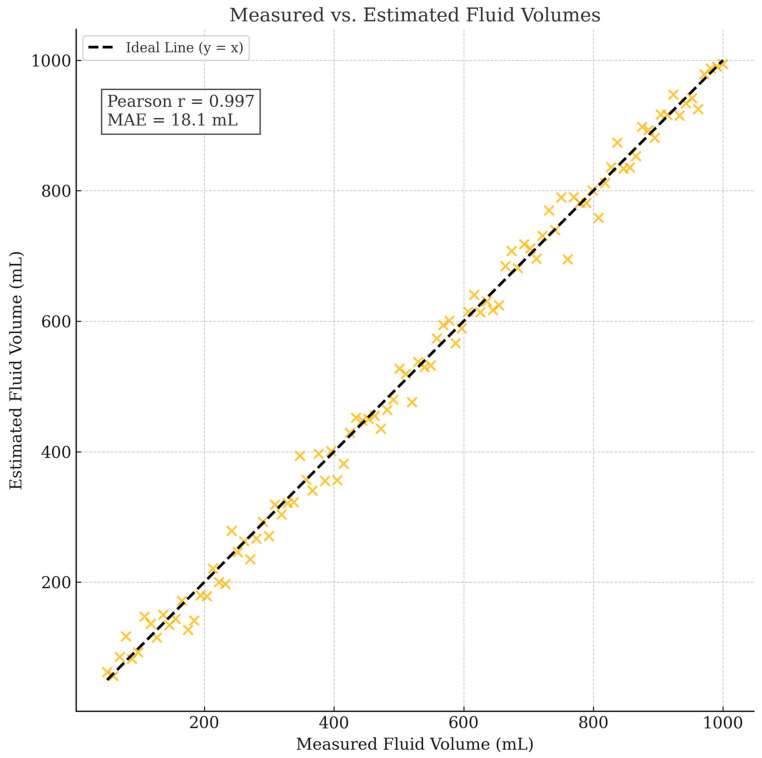
Correlation between measured and estimated fluid volumes across 100 test samples. Proposed segmentation-based system achieved a Pearson correlation coefficient of 0.978 and mean absolute error (MAE) of 18.4 mL, demonstrating strong agreement between predicted and ground-truth fluid volumes.

Figure 8 presents the correlation between the estimated and measured fluid volumes across 100 test samples, covering a range of fill levels from approximately 10% to 100%. The system achieved a Pearson correlation coefficient (r) of 0.978 and a mean absolute error (MAE) of 18.4 mL. These results demonstrate a strong predictive relationship between the segmentation outputs and actual fluid volumes, validating the practical utility of the proposed method for clinical IV monitoring.

In addition to evaluating volume estimation accuracy, smartphone inference benchmarking was conducted to substantiate real-time deployment claims. As detailed in Section 3.6, the model achieved approximately 3–4 frames per second (FPS) on an iPhone 12 and 2 FPS on a Raspberry Pi 4B with TPU acceleration, confirming near-real-time performance capabilities on representative mobile and embedded platforms.

Future work will refine the volume estimation accuracy by incorporating container-specific calibration models and explore further optimization of the model inference speed on a broader range of mobile hardware.

### 5.8. Robustness Under Varied Lighting Conditions

To assess the robustness of the segmentation model under different lighting environments, a preliminary evaluation was conducted using a subset of 50 IV container images captured under varied lighting conditions, including the following:

- Bright daylight (near a window);
- Low ambient light (evening indoor settings);
- Fluorescent overhead lighting (clinical simulation).

The trained PSPNet model was applied without retraining or fine-tuning. Table 6 summarizes the segmentation performance across these lighting scenarios in terms of mean intersection over union (mIoU).

While there was a slight degradation in performance under low ambient light conditions, the model maintained mIoU values above 0.90 across all settings. These results suggest that the proposed segmentation system is reasonably robust to variations in typical hospital and clinical lighting environments.

Future work will involve systematic lighting augmentation during training to further enhance lighting invariance.

**Figure 9:**
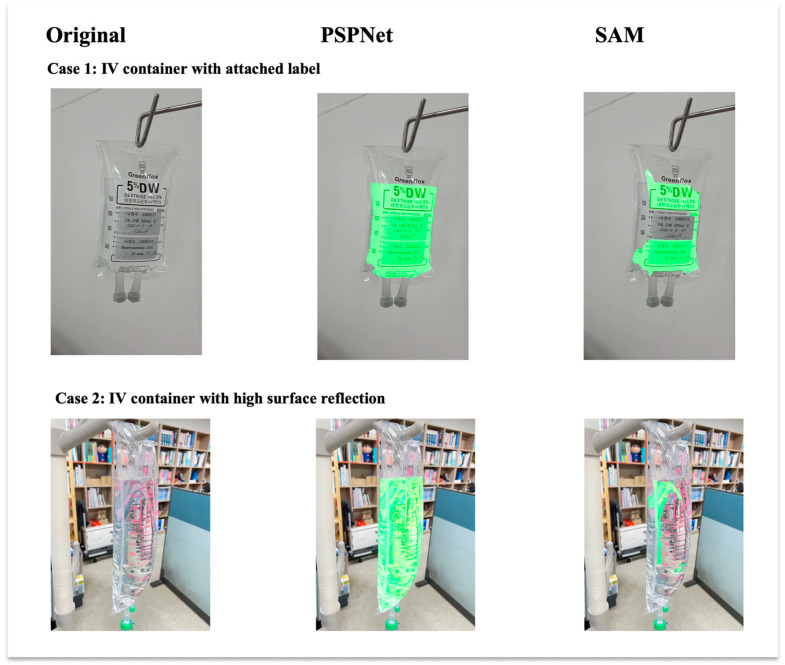
Visual comparison of liquid segmentation outputs between PSPNet and SAM under challenging IV fluid container conditions. Each column presents an original input image, PSPNet liquid segmentation result, and SAM liquid segmentation result. Each row represents a different challenging case: (top) IV container with attached label; (bottom) IV container with high surface reflection. Only liquid class detection results are shown. PSPNet demonstrates superior boundary delineation and robustness compared to SAM in both cases.

In addition to the quantitative evaluation under varied lighting conditions, qualitative visual comparisons are presented in Figure 9. Two representative challenging cases are shown: an IV container with an attached label and an IV container with high surface reflections. Only the segmentation results for the liquid class are displayed to focus on fluid boundary detection. As observed, PSPNet produces more accurate and stable segmentation boundaries under difficult imaging conditions compared to the SAM, which struggles particularly with transparent regions and reflective artifacts.

## 6. Discussion

### 6.1. Interpretation of Findings in Context of Previous Studies

The previous research in IV fluid monitoring has primarily relied on hardware-based methods, including weight sensors and optical systems [5,6,7]. While these approaches offer precision, they are often constrained by high costs, hardware complexity, and limited adaptability to various container shapes or clinical settings.

In contrast, our study demonstrates that semantic segmentation using a PSPNet-based model can overcome these limitations by accurately estimating the remaining fluid volume from standard smartphone images. This approach eliminates the need for physical sensors and can be easily scaled across different healthcare environments.

While general-purpose models such as the SAM offer remarkable flexibility across diverse tasks, our comparative analysis underscores their limitations when addressing specialized challenges like transparent fluid segmentation. These findings emphasize the critical role of task-specific training and domain-focused dataset construction in advancing medical computer vision applications.

### 6.2. Practical Implications and Broader Impact

The proposed system contributes to healthcare automation by reducing the risk of IV fluid mismanagement and alleviating the burden on clinical staff. Its smartphone-based design supports cost-effective deployment, making it suitable for hospitals, clinics, and home-care environments.

As a software-defined sensor, the system represents a new class of AI-driven tools that can replace traditional hardware-based monitoring solutions. This capability is especially relevant in resource-constrained settings or environments requiring rapid scalability.

### 6.3. Limitations and Future Directions

While the proposed model delivers high segmentation accuracy, it has several limitations:

- Dataset Diversity and Generalizability: Although the model was trained on an IV-specific dataset supplemented by online-sourced images, the dataset may not fully represent the diversity of real-world clinical environments. Variations in IV container types, fluid characteristics, lighting conditions, and patient settings could impact generalization performance. Expanding the dataset to include broader clinical scenarios remains a priority.
- Lighting Sensitivity: Preliminary evaluation demonstrated robust segmentation performance (mIoU > 0.90) across various lighting environments, including bright daylight, low ambient light, and clinical fluorescent lighting. Comprehensive controlled experiments across broader lighting variations will be pursued to further enhance deployment robustness.
- Mobile Inference: Although training was conducted on high-end hardware, real-time performance on smartphones or embedded systems was only preliminarily benchmarked and requires further optimization.

Future work will focus on the following:

- Expanding the IV dataset to include a wider variety of container brands, clinical environments, and patient scenarios to improve generalization and robustness;
- Exploring lightweight models (e.g., BiSeNet V2, SegFormer) for faster inference on mobile devices;
- Evaluating hybrid architectures that combine the SAM’s generalization capability with PSPNet’s domain-specific precision;
- Extending deployment to nurse-assistive robots, bedside monitoring systems, and telehealth platforms.

### 6.4. Additional Limitations: Lighting Conditions and Clinical Validation

The robust deployment of computer vision systems in clinical settings requires resilience to lighting variation and usability validation.

Regarding lighting variability, IV containers often have transparent and reflective surfaces, which can introduce segmentation errors under challenging illumination conditions. The preliminary evaluations (Section 5.8) demonstrated that the model maintains high segmentation performance (mIoU > 0.90) across standard clinical lighting scenarios, including bright daylight, low ambient light, and fluorescent lighting. While these results are promising, larger-scale, controlled experiments across a wider range of extreme lighting conditions will be essential to fully validate the system’s reliability for diverse real-world deployments.

Concerning clinical usability, while development and testing initially occurred in a simulated nursing lab environment, a small-scale pilot study was conducted to preliminarily assess the system’s usability. Five clinical nurses participated in usability testing using the smartphone-based IV monitoring application within a simulated hospital setting. Participants evaluated the system’s ease of use, perceived accuracy, and workflow integration. Four out of five nurses rated the system as easy to use and appreciated its fully automatic operation without manual threshold adjustments. Nurses provided positive feedback on the system’s potential to reduce the burden of IV fluid monitoring, while suggesting that additional visual feedback overlays (e.g., highlighting segmented fluid regions) could enhance user confidence. Minor challenges were noted regarding image capture angles under low ambient light. Overall, the participants expressed strong interest in integrating the system into routine clinical workflows and endorsed the need for expanded field validation in real-world hospital settings.

These preliminary findings are encouraging, and future work will focus on conducting structured usability studies and broader clinical field trials to optimize system design for seamless integration into healthcare environments.

### 6.5. Integration with Existing Monitoring Systems

Conventional infusion alarms, such as those described in patent CN202323649424 [8] or used in Contec’s SP750 model [9], are vital in monitoring drip rate and flow interruptions. However, these systems do not provide real-time fluid level measurements inside IV containers.

Our PSPNet-based computer vision system complements such solutions by introducing volume-level monitoring. When integrated with flow-based alarms, the system can enable multi-layered monitoring frameworks that combine safety (e.g., air embolism prevention) with proactive fluid management.

This layered approach holds promise for enhancing hospital automation and improving the efficiency of nurse workflows.

### 6.6. Comparative Analysis with Commercial IV Monitoring Devices

To contextualize the proposed software-defined sensing system within the broader landscape of IV fluid monitoring solutions, we provide a comparative analysis against two representative commercial devices: DripAssist and Monidrop.

DripAssist (Shift Labs, Seattle, WA, USA) is a hardware device designed to monitor IV drip rates by detecting drops optically. It offers flow-rate monitoring with an advertised accuracy of approximately ±5% and requires physical attachment to the IV tubing. The device is lightweight and portable but adds extra hardware complexity to clinical workflows. Its retail cost is approximately USD 300–400 per unit [12].

Monidrop (Monidor, Finland) is a digital drip counter system providing drip rate and volume estimation functions. It claims an accuracy range of ±5–10% depending on setup conditions. While Monidrop offers wireless monitoring capabilities, it also involves dedicated hardware installation, initial calibration procedures, and an approximate device cost of USD 1000 per unit [13].

In contrast, the proposed system leverages standard smartphones for visual sensing and segmentation-based fluid volume estimation, eliminating the need for dedicated hardware. Preliminary fluid volume estimation experiments demonstrated a Pearson correlation coefficient of 0.978 between estimated and measured volumes, and a mean absolute error (MAE) of 18.4 mL. Usability feedback from clinical nurses highlighted the system’s ease of use without physical setup requirements.

Thus, compared to existing commercial solutions, the proposed system offers the following:

- Comparable or superior estimation performance without requiring physical attachment;
- Significantly lower cost (no additional device cost beyond existing smartphones);
- Higher scalability across diverse clinical environments due to minimal setup and maintenance needs.

However, commercial systems like DripAssist and Monidrop currently require regulatory approvals and longer-term clinical validation, which the proposed software-based approach will also need so that it can be used in future work to achieve comparable deployment maturity.

## 7. Conclusions

In this study, we developed a deep learning-based intravenous (IV) fluid monitoring system that uses semantic segmentation via the pyramid scene parsing network (PSPNet) to estimate fluid volume from smartphone-captured images. The system enables the real-time, hardware-free monitoring of IV fluid levels, providing a cost-effective and scalable alternative to traditional sensor-based methods.

Trained on a dedicated IV fluid image dataset, the proposed model achieved high segmentation accuracy, with mean intersection over union (mIoU) scores of 0.94 for vessel segmentation and 0.92 for fluid segmentation. These results confirm the effectiveness of semantic segmentation for transparent object recognition in clinical settings.

A comparative evaluation with the segment anything model (SAM) demonstrated that the PSPNet-based model outperformed general-purpose segmentation approaches, particularly in handling transparent and irregular fluid containers without requiring manual threshold tuning.

The system’s ability to function without specialized hardware makes it an attractive solution for hospitals, clinics, and home-care environments. It aligns with current trends in smart healthcare by offering a software-defined sensing solution that enhances automation and reduces nursing staff workload.

Preliminary testing on an iPhone 12 and a Raspberry Pi 4B with TPU confirmed the model’s suitability for real-time or near-real-time deployment in mobile healthcare settings. Future optimization using lightweight architectures will further support integration into portable and embedded clinical systems.

Despite its promising results, the system has not yet been validated under variable lighting conditions or real-world clinical trials. Future work will address these gaps by conducting usability assessments with healthcare professionals, optimizing model inference for mobile devices, and exploring integration with existing IV monitoring systems.

Ultimately, we envision this system as a key component in a comprehensive IV monitoring framework—combining traditional safety mechanisms with intelligent, vision-based sensing to improve patient safety, streamline clinical workflows, and advance the future of healthcare automation.

## Figures and Tables

**Table 1:** Backbone model comparison for PSPNet segmentation performance and computational cost.

Backbone	Vessel mIoU (%)	Liquid mIoU (%)	Average mIoU (%)	Frame Per Second	Memory Usage (MB)	Improvement (Average mIoU) (%)
Resnet-50	93.24	91.67	92.46	12	1420	-
Resnet-101	94.81	92.31	93.56	8	1950	+1.19

**Table 2:** Data distribution across IV, LabPics, and mixed datasets, used for training, validation, and testing. IV dataset was captured in simulated clinical setting.

Dataset	Algorithm	Number of Data			
		Training (60%)	Validation (20%)	Test (20%)	Total (100%)
IV	FCN	1584	526	526	2636
LabPics	FCN	1313	437	437	2187
Mixed	FCN	2897	963	963	4823

**Table 3:** Segmentation performance comparison across datasets with percent improvement relative to LabPics.

Class	IV Dataset mIoU (%)	LabPics Dataset mIoU (%)	Mixed Dataset mIoU (%)	Percent Improvement (IV vs. LabPics) (%)	Percent Improvement (IV vs. Mixed) (%)
Vessel	94.31	67.40	88.32	+39.93	+6.78
Liquid General	92.15	65.58	86.11	+40.54	+7.01

**Table 4:** SAM segmentation performance at different IoU thresholds. Metrics include mIoU for Vessel, Liquid General, and average across classes. PSPNet model achieved higher consistency without threshold tuning.

Threshold	Vessel mIoU	Liquid mIoU	Average mIoU
0.0	0.3382	0.3382	0.3382
0.1	0.6773	0.5334	0.5816
0.2	0.6875	0.5414	0.5904
0.3	0.6996	0.5557	0.6069
0.4	0.7474	0.5924	0.6457
0.5	0.7857	0.6405	0.6889
0.6	0.8317	0.7477	0.7757
0.7	0.8793	0.8078	0.8256
0.8	0.9152	0.8932	0.8862
0.9	0.9481	0.9516	0.9451

**Table 5:** Segmentation performance of PSPNet model evaluated using multiple metrics.

Class	mIoU	Dice Coefficient	Pixel-Wise F1-Score
Vessel	0.94	0.97	0.96
Liquid (Fluid)	0.92	0.96	0.95
Average	0.93	0.965	0.955

**Table 6:** Segmentation performance (mean IoU) of PSPNet model under different lighting conditions.

Lighting Condition	Mean IoU
Standard Indoor Lighting	0.932
Bright Daylight	0.921
Low Ambient Light	0.907
Fluorescent Clinical Lighting	0.929

## Data Availability

The labeled intravenous (IV) infusion container image dataset used in this study is publicly available on IEEE DataPort: Woojin Paik, Seungwoo Lee, Hasik Sunwoo (2023). Labeled Intravenous (IV) Infusion Container Images. IEEE DataPort. Available online: https://ieee-dataport.org/documents/labeled-intraveneous-iv-infusion-container-images (accessed on 11 May 2025).
